# Health-Related Quality of Life and Psychological Distress Status in Survivors of Nasopharyngeal Carcinoma: A Retrospective Study

**DOI:** 10.7759/cureus.71070

**Published:** 2024-10-08

**Authors:** Nawaf S Alghamdi, Almoaidbellah Rammal, Reem M Alzahrani, Alwaleed H Kaneetah, Anas K Alghamdi, Abdulelah A Alnahdi, Amjad A Shawly, Raghad A Alzahrani

**Affiliations:** 1 College of Medicine, King Abdulaziz University, Jeddah, SAU; 2 Otolaryngology - Head and Neck Surgery, King Abdulaziz University, Jeddah, SAU

**Keywords:** anxiety, depression, health related quality of life, nasopharyngeal cancer, npc, psychological distress, psychological impact, quality of life

## Abstract

Introduction

Nasopharyngeal carcinoma (NPC) is an uncommon cancer globally, with a low incidence in Western countries. However, this rate increases significantly in some endemic regions, including the Middle East. Although studies have evaluated the physical health outcomes, treatment efficacy, and short-term effects, long-term effects - especially quality of life (QoL) - have been neglected. Therefore, we aimed to assess the QoL and psychological distress status of survivors of NPC and investigate the demographic, clinical, temporal, and therapeutic factors that impact the well-being of such patients.

Methodology

This cross-sectional analysis was performed in 2023 at the King Abdulaziz University Hospital (KAUH), a major tertiary care facility located in Jeddah, Kingdom of Saudi Arabia. We retrospectively reviewed all reported cases of NPC at KAUH from 2010 to 2023 and identified 34 patients who met our inclusion criteria. Data on the QoL and psychological distress status were collected using telephone interviews, during which three questionnaires were administered: the “European Organization for Research and Treatment of Cancer Quality of Life Questionnaire Core 30 Version 3.0” (EORTC QLQ-C30), the “European Organization for Research and Treatment of Cancer Quality of Life Questionnaire - Head and Neck Module 43” (EORTC QLQ-HN43), an additional questionnaire designed to be utilized alongside the EORTC QLQ-C30, and the “Hospital Anxiety and Depression Scale.” Demographic and clinical information were also obtained.

Results

The patients had a mean age of 49.88 ± 2.27 years (range: 18-70 years). The mean survival duration was 5.88 years (range: 1-14 years). From the pooled data, abnormal borderline depression, borderline anxiety, and anxiety were identified in five patients (14.7%), four patients (11.76%), and two patients (5.88%), respectively. Patient age showed a significant positive correlation with both “fatigue” (r = 0.367, p = 0.033) and “social contact” (r = 0.423, p = 0.013), where higher scores on both symptom scales indicate worse symptoms (i.e., greater fatigue and more difficulties with social interactions). The mean “global health status/QoL” was significantly lower in patients with comorbidities (p = 0.039) and “oral dryness” (p = 0.027) compared to those without. The tumor stage showed a significant negative correlation with the “global health status/QoL” (r = -0.664, p = 0.001).

Conclusion

A significant number of survivors of NPC experienced borderline depression and anxiety. “Global health status/QoL” was low in advanced tumor stages. The mean “global health status/QoL” was low in the presence of certain factors, including coexisting comorbidities and treatment-related side effects. Our findings suggest that patients diagnosed with NPC should undergo assessments for anxiety and depression before, during, and after treatment. Moreover, symptomatic treatment of therapeutic side effects, especially “oral dryness,” should be undertaken. “Oral dryness,” experienced by most of the patients, was significantly correlated with a low mean “global health status/QoL.”

## Introduction

Nasopharyngeal carcinoma (NPC) is an uncommon cancer across the globe [1], with an annual incidence of fewer than 1 per 100,000 individuals in Western countries. However, this rate increases to 20-50 per 100,000 individuals in some endemic regions, including Southern China, Southeast Asia, the Middle East, and North Africa [2]. In Saudi Arabia, approximately 110,075 cases of NPC were identified from 2007 to 2016, with NPC considered the leading type of head and neck cancer (35%) and the 17th most prevalent cancer (2.9%) [3,4]. The exact etiology of NPC remains uncertain, although potential risk factors encompass a familial predisposition to NPC, Epstein-Barr virus infection, and the consumption of high-salt meats and fish [5]. NPC may not manifest clinically in the initial stages; however, as it progresses to more advanced stages, its clinical manifestations include enlarged neck lymph nodes, nasal and salivary bleeding, hearing impairment, and ear infections [6].

Research on the quality of life (QoL) and psychological distress status of survivors of NPC in the Middle East, especially in Saudi Arabia, is exceedingly scarce. In addition, the existing literature focuses on physical health outcomes, treatment efficacy, and short-term effects, neglecting the importance of long-term outcomes and mental health effects. Therefore, we aimed to assess the QoL and psychological distress status of survivors of NPC and to investigate the demographic, clinical, temporal, and therapeutic factors that impact the well-being of such patients.

## Materials and methods

Study design, setting, and population

This cross-sectional analysis was performed in 2023 at the King Abdulaziz University Hospital (KAUH), a major tertiary care facility located in Jeddah, Kingdom of Saudi Arabia (KSA). We retrospectively reviewed all NPC cases reported from 2010 to 2023 and identified 152 confirmed cases. We included patients who had completed cancer treatment for one year or more, were aware of their cancer diagnosis, and were at least 18 years of age. The exclusion criteria were patients with recurrence or metastasis, or those diagnosed with psychiatric disorders and/or prescribed psychiatric medications. Among the 152 patients, 55 had died (36.2%), 52 had lost contact with the hospital and were unreachable by telephone (34.2%), six did not fulfill the eligibility criteria (3.9%), and five declined to participate (3.3%). Ultimately, the study included 34 patients, representing 22.4% of the initial cohort.

Data collection method

We conducted telephone interviews with patients to obtain their demographic information, including their sex, nationality, marital status, household income, and the presence of comorbidities, including diabetes and/or hypertension.

We reviewed the patients' electronic records to collect data regarding the date of birth and clinical variables, including the date of cancer diagnosis, survival duration, pathological diagnosis of squamous cell carcinoma versus undifferentiated cancer, tumor-node-metastasis (TNM) staging, and the modality of treatment provided to patients (including chemotherapy, radiotherapy, and/or surgery). Staging of cancer was performed according to the “Cancer Staging Manual” by the “American Joint Committee on Cancer,” eighth edition. Among the 34 included patients, TNM and cancer staging data were missing for 13 patients (38.2%); pathology data were missing for seven patients (20.6%); and modality of treatment data were missing for one patient (2.9%).

Data on patient QoL and psychological distress status were collected through telephone interviews using three questionnaires; we utilized the Arabic versions of all three. The first questionnaire was the “European Organization for Research and Treatment of Cancer Quality of Life Questionnaire Core 30 Version 3.0” (EORTC QLQ-C30), which is used to assess the QoL of patients with cancer. Permission to use this tool was granted following registration approval. This questionnaire has 30 items, with 28 items scored on a scale of 1-4 and two items on a scale of 1-7. These items include the following scales: “global health status/QoL”; five functional scales that assess “physical functioning,” “role functioning,” “emotional functioning,” “cognitive functioning,” and “social functioning”; three symptom scales that evaluate “fatigue,” “nausea and vomiting,” and “pain”; and six single-item measures of “dyspnea,” “insomnia,” “appetite loss,” “constipation,” “diarrhea,” and “financial difficulties.” The scores for each of the scales and single items ranged from 0 to 100, with higher scores for the “global health status/QoL,” functional scales, symptom scales, and single-item measures indicating a higher QoL, level of functioning, and grade of symptoms.

The second questionnaire was the “European Organization for Research and Treatment of Cancer Quality of Life Questionnaire - Head and Neck Module 43” (EORTC QLQ-HN43), an additional questionnaire designed to assess the QoL in patients with head and neck cancer and to be utilized alongside the EORTC QLQ-C30. Permission to use this tool was granted following registration approval. This questionnaire has 43 items scored from 1 to 4, 12 multi-item scales, and seven single-item measures. The multi-item scales include assessments of “pain in the mouth,” “swallowing,” “problems with teeth,” “dry mouth and sticky saliva,” “problems with senses,” “speech,” “body image,” “social eating,” “sexuality,” “problems with shoulder,” “skin problems,” and “fear of progression.” Single-item measures include assessments of “problems opening mouth,” “coughing,” “social contact,” “swelling in the neck,” “weight loss,” “problems with wound healing,” and “neurological problems.” The scores for each multi-item scale and single-item measure ranged from 0 to 100, with higher scores signifying increased severity of symptoms or problems.

The third questionnaire was the “Hospital Anxiety and Depression Scale” (HADS) [7,8], which assesses anxiety and depression. It is a 14-item questionnaire scored from 0 to 3, with seven items in each of the anxiety and depression subscales. Total scores on the anxiety and depression subscales range from 0 to 21.

Following the interview, we inquired about patients’ symptoms resulting from treatment, including headache, hearing loss, visual disturbances, difficulty swallowing, oral dryness, mouth ulcers, dry nose, nasal congestion, and fatigue [9-11]. We also inquired about persistent fears of disease recurrence.

Statistical analysis

We used Microsoft Excel 2019 (Microsoft® Corp., Redmond, WA, USA) for data organization and IBM SPSS Statistics for Windows, Version 21 (Released 2012; IBM Corp., Armonk, NY, USA) for statistical analysis. Continuous variables are presented using means and standard deviations, while categorical variables are presented using frequencies and percentages. “Student’s t-test” and the “Chi-squared test” were used to assess differences between continuous and categorical variables, respectively. Significance was defined as p < 0.05.

Ethical approval

This study was conducted following the approval of the Institutional Review Board at KAUH and the ethical clearance provided by the Research Ethics Committee of KAUH (Ref: 201-23). All participants gave verbal consent prior to the interviews and were informed about the study’s purpose.

## Results

The patients’ demographic characteristics are presented in Table 1. The patients had a mean age of 49.88 ± 2.27 years (range: 18-70 years). The survival duration ranged from 1 to 14 years, with a mean of 5.88 years. Men were more affected by NPC than women (20 men, 58.8% vs. 14 women, 41.2%), and non-Saudi patients were more affected by NPC than Saudi patients (18 non-Saudi patients, 52.9% and 16 Saudi patients, 47.1%). Most participants were married, with 24 patients (70.6%) being married, while 18 patients (52.9%) reported a household income of ≤5,000 SR. Comorbidities were reported in 12 patients (35.3%), six of whom had diabetes mellitus (17.6%), while 11 (32.4%) had hypertension.

**Table 1:** Demographic characteristics of patients (n = 34). ^a^Mean ± standard deviation (range) or n (%)

Characteristics	Value^a^
Age (years)	49.88 ± 2.27 (18-70)
Survival duration (years)	5.88 ± 3.53 (1-14)
Sex	
Male	20 (58.8%)
Female	14 (41.2%)
Nationality	
Saudi	16 (47.1%)
Non-Saudi	18 (52.9%)
Marital status	
Single	7 (20.6%)
Married	24 (70.6%)
Divorced	1 (2.9%)
Widowed	2 (5.9%)
Household income	
≤5,000 SR	18 (52.9%)
5,001-10,000 SR	9 (26.5%)
10,001-20,000 SR	5 (14.7%)
20,001-30,000 SR	1 (2.9%)
>30,000 SR	1 (2.9%)
Comorbidities	
No	22 (64.7%)
Yes	12 (35.3%)
Diabetes mellitus	6 (17.6%)
Hypertension	11 (32.4%)

The symptoms that NPC survivors are currently experiencing include oral dryness in 28 patients (82.4%), hearing loss in 25 patients (73.5%), difficulty swallowing in 19 patients (55.9%), fatigue in 17 patients (50.0%), visual disturbances in 17 patients (50.0%), dry nose in 15 patients (44.1%), nasal congestion in 13 patients (38.2%), headache in 10 patients (29.4%), persistent fears of disease recurrence in nine patients (26.5%), and mouth ulcers in three patients (8.8%). Pathological examinations revealed undifferentiated carcinomas in 21 (77.8%) cases and squamous cell carcinomas in six (22.2%) cases. The cases were T4 in nine patients (42.9%), N2 in 13 patients (61.9%), and stage IV in 12 patients (57.1%). Treatments included chemotherapy and radiotherapy in 22 patients (66.7%) and chemotherapy, radiotherapy, and surgery in 11 patients (33.3%) (Table 2).

**Table 2:** Clinical characteristics of patients. ^a^n (%) TNM: Tumor-node-metastasis

Characteristics	Frequency	Value^a^
Symptoms	(n = 34)	
Oral dryness		28 (82.4%)
Hearing loss		25 (73.5%)
Difficulty swallowing		19 (55.9%)
Fatigue		17 (50.0%)
Visual disturbances		17 (50.0%)
Dry nose		15 (44.1%)
Nasal congestion		13 (38.2%)
Headache		10 (29.4%)
Persistent fears of disease recurrence		9 (26.5%)
Mouth ulcers		3 (8.8%)
Pathology	(n = 27), 7 patients had missing data	
Squamous cell carcinoma		6 (22.2%)
Undifferentiated carcinoma		21 (77.8%)
TNM	(n = 21), 13 patients had missing data	
Tumor grade (T)		
1		3 (14.3%)
2		4 (19.0%)
3		5 (23.8%)
4		9 (42.9%)
Nodes (N)		
0		2 (9.5%)
1		1 (4.8%)
2		13 (61.9%)
3		5 (23.8%)
Stage	(n = 21), 13 patients had missing data	
II		1 (4.8%)
III		8 (38.1%)
IV		12 (57.1%)
Type of treatment	(n = 33), 1 patient had missing data	
Chemotherapy + radiation		22 (66.7%)
Chemotherapy + radiation + surgery		11 (33.3%)
Radiotherapy		33 (100.0%)
Chemotherapy		33 (100.0%)
Surgery		11 (33.3%)

The HADS, EORTC QLQ-C30, and EORTC QLQ-HN43 scores are shown in Table 3. The mean scores for depression and anxiety were 3.38 and 3.97, respectively. Five (14.7%) patients had abnormal borderline depression, four (11.8%) had borderline anxiety, and two (5.9%) had anxiety. The mean score for “global health status/QoL” was 63.97 ± 22.54. The highest mean functioning EORTC QLQ-C30 score was for “role functioning” (81.37 ± 29.23), followed by “emotional functioning” (80.39 ± 20.40), “physical functioning” (78.43 ± 20.24), “cognitive functioning” (75.00 ± 26.35), and “social functioning” (75.00 ± 27.60). The highest EORTC QLQ-C30 symptom score was for “fatigue” (25.49 ± 22.81), and the lowest was for “diarrhea” (4.90 ± 18.59). The highest QLQ-HN43 symptom score was for “dry mouth and sticky saliva” (42.16 ± 23.65), and the lowest was for “coughing” (4.90 ± 14.52).

**Table 3:** HADS, EORTC QLQ-C30, and EORTC QLQ-HN43 scores of patients (n = 34). ^a^Higher scores indicate better conditions; ^b^Higher scores indicate greater symptoms HADS: Hospital Anxiety and Depression Scale; EORTC QLQ-C30: European Organization for Research and Treatment of Cancer Quality of Life Questionnaire Core 30 Version 3.0; EORTC QLQ-HN43: European Organization for Research and Treatment of Cancer Quality of Life Questionnaire Head and Neck Module; QoL: Quality of life

Scales	Mean ± standard deviation or n (%)	Minimum-maximum	95% confidence interval
HADS scores			
Depression total score	3.38 ± 3.02	0.00-10.00	2.33-4.43
Depression category			
Normal (0-7)	29 (85.3%)		
Borderline abnormal (8–10)	5 (14.7%)		
Anxiety total score	3.97 ± 3.42	0-11	2.78-5.16
Anxiety category			
Normal (0-7)	28 (82.4%)		
Borderline abnormal (8-10)	4 (11.8%)		
Abnormal (case) (11-21)	2 (5.9%)		
QLQ-C30 scores			
Global health status/QoL^a^	63.97 ± 22.54	8.33-100.00	56.11-71.84
Functioning^a^			
Physical functioning	78.43 ± 20.24	33.33-100.00	71.37-85.49
Role functioning	81.37 ± 29.23	16.67-100.00	71.17-91.57
Emotional functioning	80.39 ± 20.40	25.00-100.00	73.27-87.51
Cognitive functioning	75.00 ± 26.35	16.67-100.00	65.81-84.19
Social functioning	75.00 ± 27.60	33.33-100.00	65.37-84.63
Symptoms^b^			
Fatigue	25.49 ± 22.81	0.00-77.78	17.53-33.45
Nausea and vomiting	7.35 ± 13.73	0.00-50.00	2.56-12.14
Pain	23.04 ± 24.95	0.00-83.33	14.33-31.74
Dyspnea	17.65 ± 28.70	0.00-100.00	7.63-27.66
Insomnia	22.55 ± 31.48	0.00-100.00	11.56-33.53
Appetite loss	17.65 ± 29.85	0.00-100.00	7.23-28.06
Constipation	20.59 ± 29.60	0.00-100.00	10.26-30.92
Diarrhea	4.90 ± 18.59	0.00-100.00	-1.58-11.39
Financial difficulties	19.61 ± 32.94	0.00-100.00	8.11-31.10
QLQ-HN43 scores^b^			
Pain in the mouth	16.91 ± 16.60	0.00-66.67	11.12-22.70
Swallowing	21.57 ± 18.59	0.00-66.67	15.08-28.06
Problems with teeth	39.87 ± 28.04	0.00-100.00	30.08-49.65
Dry mouth and sticky saliva	42.16 ± 23.65	0.00-83.33	33.90-50.41
Problems with senses	21.08 ± 27.62	0.00-100.00	11.44-30.71
Speech	26.67 ± 25.00	0.00-80.00	17.94-35.39
Body image	13.73 ± 21.72	0.00-77.78	6.15-21.30
Social eating	17.89 ± 22.39	0.00-66.67	10.08-25.71
Sexuality	22.55 ± 35.74	0.00-100.00	10.08-35.02
Problems with shoulder	21.08 ± 29.39	0.00-100.00	10.82-31.33
Skin problems	14.05 ± 15.06	0.00-55.56	8.80-19.31
Fear of progression	25.49 ± 26.98	0.00-83.33	16.08-34.90
Problems opening mouth	41.18 ± 37.66	0.00-100.00	28.04-54.32
Coughing	4.90 ± 14.52	0.00-66.67	-0.17-9.97
Social contact	13.73 ± 27.36	0.00-100.00	4.18-23.27
Swelling in the neck	15.69 ± 29.85	0.00-100.00	5.27-26.10
Weight loss	32.35 ± 34.31	0.00-100.00	20.38-44.33
Problems with wound healing	7.84 ± 20.20	0.00-66.67	0.80-14.89
Neurological problems	32.35 ± 32.29	0.00-100.00	21.09-43.62

Patients with comorbidities had a significantly lower mean score for “global health status/QoL” compared to those without (p = 0.039). Additionally, patients with “oral dryness” had a significantly lower mean score for “global health status/QoL” than those without (p = 0.027) (Table 4).

**Table 4:** Global health status/QoL score in relation to demographic and clinical characteristics of patients. * p<0.05 EORTC QLQ-C30: European Organization for Research and Treatment of Cancer Quality of Life Questionnaire Core 30 Version 3.0; QoL: Quality of life

Items	Mean ± standard deviation	95% confidence interval	Significance
Sex			0.257
Male	60.00 ± 24.42	48.57-71.43	
Female	69.64 ± 19.95	58.70-80.58	
Nationality			0.364
Saudi	67.71 ± 25.80	53.96-81.45	
Non-Saudi	60.65 ± 19.34	51.03-70.27	
Marital status			0.118
Single	79.76 ± 20.33	60.96-98.57	
Married	58.33 ± 22.25	48.94-67.73	
Divorced	66.67 ± 0.00	-	
Widowed	75.00 ± 11.79	-211.78	
Household income			0.102
≤5,000 SR	55.56 ± 17.85	46.68-64.43	
5,001-10,000 SR	72.22 ± 27.95	50.74-93.71	
10,001-20,000 SR	71.67 ± 20.07	46.75-96.59	
20,001-30,000 SR	66.67 ± 0.00	-	
>30,000 SR	100.00 ± 0.00	-	
Comorbidities			0.039*
No	70.45 ± 20.04	61.57-79.34	
Yes	52.08 ± 22.79	37.61-66.56	
Symptoms			
Oral dryness	59.82 ± 20.92	51.71-67.93	0.027*
Hearing loss	63.33 ± 22.82	53.91-72.75	0.828
Difficulty swallowing	65.79 ± 24.04	54.20-77.38	0.38
Fatigue	59.31 ± 22.79	47.59-71.04	0.413
Vision disturbance	57.35 ± 21.83	46.03-68.58	0.084
Dry nose	62.78 ± 24.17	49.40-76.16	0.93
Nasal congestion	60.26 ± 24.80	45.27-75.24	0.591
Headache	63.33 ± 21.22	48.15-78.52	0.863
Persistent fears of disease recurrence	56.48 ± 24.57	37.59-75.37	0.374
Mouth ulcers	66.67 ± 16.67	25.26-108.07	0.83

A comparison of scores for EORTC QLQ-C30 and EORTC QLQ-HN43 according to the type of treatment showed no significant differences between patients who received chemotherapy and radiotherapy, versus those treated with chemotherapy, radiotherapy, and surgery (Table 5).

**Table 5:** Comparison of EORTC QLQ-C30 and EORTC QLQ-HN43 scores according to the type of treatment. EORTC QLQ-C30: European Organization for Research and Treatment of Cancer Quality of Life Questionnaire Core 30 Version 3.0; EORTC QLQ-HN43: European Organization for Research and Treatment of Cancer Quality of Life Questionnaire Head and Neck Module; QoL: Quality of life

Domain	Chemotherapy + radiation (n = 22)	Chemotherapy + radiation + surgery (n = 11)	Significance
QLQ-C30 scores			
Global health status/QoL	64.77 ± 23.28	63.64 ± 22.75	0.892
Functioning			
Physical functioning	77.88 ± 21.42	79.39 ± 19.65	0.954
Role functioning	83.33 ± 28.17	75.76 ± 32.80	0.45
Emotional functioning	85.23 ± 18.89	71.21 ± 21.85	0.053
Cognitive functioning	76.52 ± 29.39	69.70 ± 19.46	0.213
Social functioning	71.97 ± 28.82	84.85 ± 21.67	0.268
Symptoms			
Fatigue	26.77 ± 22.13	25.25 ± 24.89	0.771
Nausea and vomiting	6.06 ± 10.97	10.61 ± 18.67	0.733
Pain	25.76 ± 26.59	19.70 ± 22.13	0.55
Dyspnea	15.15 ± 26.68	24.24 ± 33.63	0.408
Insomnia	24.24 ± 31.17	21.21 ± 34.23	0.684
Appetite loss	22.73 ± 33.15	3.03 ± 10.05	0.056
Constipation	25.76 ± 34.01	12.12 ± 16.82	0.366
Diarrhea	4.55 ± 21.32	6.06 ± 13.48	0.235
Financial difficulties	25.76 ± 38.40	9.09 ± 15.57	0.361
QLQ-HN43 scores			
Pain in the mouth	17.42 ± 14.53	16.67 ± 21.41	0.545
Swallowing	18.18 ± 15.35	25.76 ± 22.81	0.405
Problems with teeth	34.85 ± 27.71	44.44 ± 23.31	0.248
Dry mouth and sticky saliva	39.39 ± 22.74	48.48 ± 26.30	0.338
Problems with senses	16.67 ± 24.67	24.24 ± 28.25	0.42
Speech	23.33 ± 24.84	28.48 ± 21.31	0.386
Body image	9.60 ± 18.87	23.23 ± 25.56	0.113
Social eating	15.91 ± 20.40	18.18 ± 24.67	0.84
Sexuality	24.24 ± 37.35	19.70 ± 35.60	0.649
Problems with shoulder	22.73 ± 30.67	19.70 ± 28.69	0.766
Skin problems	13.13 ± 14.80	15.15 ± 16.68	0.779
Fear of progression	21.21 ± 20.69	34.85 ± 36.86	0.453
Problems opening mouth	45.45 ± 33.41	36.36 ± 45.84	0.496
Coughing	6.06 ± 16.70	3.03 ± 10.05	0.686
Social contact	12.12 ± 28.26	18.18 ± 27.34	0.334
Swelling in the neck	18.18 ± 30.39	12.12 ± 30.81	0.45
Weight loss	33.33 ± 35.63	27.27 ± 32.72	0.65
Problems with wound healing	6.06 ± 16.70	12.12 ± 26.97	0.646
Neurological problems	31.82 ± 34.85	33.33 ± 29.81	0.792

Correlations between EORTC QLQ-C30 and EORTC QLQ-HN43 scores with age, survival duration, and tumor stage are shown in Table 6. Patient age had a significant negative correlation with “emotional functioning” (r = -0.454, p = 0.007) and significant positive correlations with “fatigue” (r = 0.367, p = 0.033), “nausea and vomiting” (r = 0.339, p = 0.050), “dyspnea” (r = 0.450, p = 0.008), “constipation” (r = 0.407, p = 0.017), and “social contact” (r = 0.423, p = 0.013). The survival duration had a significant negative correlation with “coughing” (r = -0.346, p = 0.045). Tumor stage showed a significant negative correlation with the “global health status/QoL” (r = -0.664, p = 0.001) and significant positive correlations with “fatigue” (r = 0.451, p = 0.040), “body image” (r = 0.446, p = 0.043), and “weight loss” (r = 0.562, p = 0.008).

**Table 6:** Correlations of age, survival duration, and tumor stage with EORTC QLQ-C30 and EORTC QLQ-HN43 scores. *p < 0.05; **p ≤ 0.01 EORTC QLQ-C30: European Organization for Research and Treatment of Cancer Quality of Life Questionnaire Core 30 Version 3.0; EORTC QLQ-HN43: European Organization for Research and Treatment of Cancer Quality of Life Questionnaire Head and Neck Module; QoL: Quality of life

Domain	Age (years)	Survival duration (years)	Tumor stage
QLQ-C30 scores			
Global health status/QoL	-0.222 (0.208)	-0.121 (0.494)	-0.664 (0.001)**
Functioning			
Physical functioning	-0.313 (0.072)	0.129 (0.467)	-0.374 (0.095)
Role functioning	-0.245 (0.163)	-0.163 (0.357)	-0.296 (0.192)
Emotional functioning	-0.454 (0.007)**	-0.297 (0.088)	-0.285 (0.211)
Cognitive functioning	-0.208 (0.238)	-0.029 (0.871)	-0.270 (0.237)
Social functioning	-0.262 (0.135)	-0.010 (0.953)	-0.080 (0.730)
Symptoms			
Fatigue	0.367 (0.033)*	0.133 (0.452)	0.451 (0.040)*
Nausea and vomiting	0.339 (0.050)*	-0.071 (0.691)	0.282 (0.215)
Pain	0.152 (0.390)	-0.122 (0.491)	0.342 (0.130)
Dyspnea	0.450 (0.008)**	-0.212 (0.229)	0.065 (0.778)
Insomnia	0.122 (0.493)	-0.073 (0.681)	0.207 (0.368)
Appetite loss	0.102 (0.566)	-0.147 (0.407)	0.126 (0.588)
Constipation	0.407 (0.017)*	-0.239 (0.174)	0.031 (0.894)
Diarrhea	-0.085 (0.633)	-0.180 (0.308)	-0.233 (0.309)
Financial difficulties	0.046 (0.796)	-0.166 (0.348)	0.063 (0.787)
QLQ-HN43 scores			
Pain in the mouth	0.298 (0.087)	0.013 (0.944)	0.231 (0.313)
Swallowing	0.136 (0.358)	0.351 (0.042)	0.058 (0.802)
Problems with teeth	0.090 (0.613)	0.199 (0.259)	0.417 (0.060)
Dry mouth and sticky saliva	0.259 (0.140)	-0.020 (0.913)	0.124 (0.593)
Problems with senses	0.076 (0.668)	0.075 (0.674)	0.351 (0.118)
Speech	-0.034 (0.850)	0.160 (0.367)	0.119 (0.606)
Body image	0.031 (0.863)	0.062 (0.729)	0.446 (0.043)*
Social eating	-0.024 (0.892)	0.298 (0.087)	0.047 (0.839)
Sexuality	0.247 (0.160)	0.023 (0.896)	0.202 (0.381)
Problems with shoulder	0.057 (0.747)	0.062 (0.729)	0.383 (0.086)
Skin problems	-0.165 (0.352)	-0.032 (0.856)	-0.188 (0.415)
Fear of progression	-0.011 (0.952)	-0.149 (0.400)	0.392 (0.078)
Problems opening mouth	0.009 (0.958)	0.125 (0.480)	0.359 (0.110)
Coughing	-0.121 (0.495)	-0.346 (0.045)*	-0.143 (0.537)
Social contact	0.423 (0.013)*	0.056 (0.754)	-0.179 (0.437)
Swelling in the neck	-0.192 (0.277)	-0.284 (0.103)	0.110 (0.636)
Weight loss	0.154 (0.383)	0.176 (0.318)	0.562 (0.008)**
Problems with wound healing	-0.040 (0.821)	0.222 (0.207)	-0.155 (0.503)
Neurological problems	0.032 (0.857)	0.124 (0.485)	0.122 (0.597)

Correlations between EORTC QLQ-HN43 scores and the EORTC QLQ-C30 functional scales, as well as “global health status/QoL” scores, are illustrated in Table 7. “Physical functioning” showed significant negative correlations with “pain in the mouth” (r = -0.466, p = 0.006), “dry mouth and sticky saliva” (r = -0.416, p = 0.014), “problems with senses” (r = -0.353, p = 0.040), “speech” (r = -0.397, p = 0.020), “sexuality” (r = -0.434, p = 0.010), and “social contact” (r = -0.452, p = 0.007). “Role functioning” had significant negative correlations with “dry mouth and sticky saliva” (r = -0.449, p = 0.008), “speech” (r = -0.474, p = 0.005), “sexuality” (r = -0.457, p = 0.007), and “neurological problems” (r = -0.340, p = 0.049). “Emotional functioning” showed significant negative correlations with “problems with teeth” (r = -0.367, p = 0.033), “dry mouth and sticky saliva” (r = -0.401, p = 0.019), “speech” (r = -0.391, p = 0.022), and “sexuality” (r = -0.347, p = 0.045). “Cognitive functioning” showed significant negative correlations with “pain in the mouth” (r = -0.487, p = 0.004), “body image” (r = -0.641, p = 0.0001), “sexuality” (r = -0.384, p = 0.025), “problems opening mouth” (r = -0.461, p = 0.006), and a significant positive correlation with “problems with shoulder” (r = 0.401, p = 0.019). “Social functioning” showed a significant negative correlation with “speech” (r = -0.383, p = 0.025). “Global health status/QoL” showed significant negative correlations with “body image” (r = -0.373, p = 0.030), “sexuality” (r = -0.361, p = 0.036), and “weight loss” (r = -0.486, p = 0.004), and a significant positive correlation with “skin problems” (r = 0.342, p = 0.048).

**Table 7:** Correlations of EORTC QLQ-HN43 scores with EORTC QLQ-C30 functional scales and global health status/QoL scores. *p < 0.05; **p ≤ 0.01; ***p ≤ 0.001 EORTC QLQ-C30: European Organization for Research and Treatment of Cancer Quality of Life Questionnaire Core 30 Version 3.0; EORTC QLQ-HN43: European Organization for Research and Treatment of Cancer Quality of Life Questionnaire Head and Neck Module; QoL: Quality of life

QLQ-HN43 scores	Physical functioning	Role functioning	Emotional functioning	Cognitive functioning	Social functioning	Global health status/QoL
Pain in the mouth	-0.466 (0.006)**	-0.272 (0.120)	-0.293 (0.092)	-0.487 (0.004)**	-0.328 (0.058)	-0.190 (0.282)
Swallowing	-0.112 (0.530)	-0.154 (0.383)	-0.321 (0.064)	-0.282 (0.106)	-0.100 (0.573)	-0.187 (0.288)
Problems with teeth	-0.246 (0.160)	-0.325 (0.061)	-0.367 (0.033)*	-0.142 (0.423)	-0.154 (0.383)	-0.251 (0.151)
Dry mouth and sticky saliva	-0.416 (0.014)*	-0.449 (0.008)**	-0.401 (0.019)*	-0.310 (0.075)	-0.259 (0.140)	-0.229 (0.193)
Problems with senses	-0.353 (0.040)*	-0.253 (0.148)	-0.110 (0.536)	-0.128 (0.469)	-0.038 (0.831)	-0.135 (0.446)
Speech	-0.397 (0.020)*	-0.474 (0.005)**	-0.391 (0.022)*	-0.298 (0.086)	-0.383 (0.025)*	-0.166 (0.348)
Body image	-0.146 (0.411)	-0.312 (0.073)	-0.310 (0.074)	-0.641 (0.0001)***	-0.141 (0.426)	-0.373 (0.030)*
Social eating	-0.204 (0.248)	-0.159 (0.368)	-0.297 (0.088)	-0.206 (0.243)	-0.142 (0.422)	-0.238 (0.175)
Sexuality	-0.434 (0.010)*	-0.457 (0.007)**	-0.347 (0.045)*	-0.384 (0.025)*	-0.326 (0.060)	-0.361 (0.036)*
Problems with shoulder	0.256 (0.144)	-0.242 (0.168)	-0.201 (0.255)	0.401 (0.019)*	-0.234 (0.183)	0.074 (0.679)
Skin problems	0.097 (0.586)	0.237 (0.117)	-0.050 (0.779)	0.187 (0.291)	0.010 (0.956)	0.342 (0.048)*
Fear of progression	-0.026 (0.886)	-0.116 (0.515)	-0.013 (0.943)	0.159 (0.370)	-0.124 (0.486)	-0.061 (0.731)
Problems opening mouth	-0.160 (0.367)	-0.295 (0.090)	-0.287 (0.099)	-0.461 (0.006)**	-0.097 (0.587)	-0.071 (0.689)
Coughing	-0.153 (0.386)	0.262 (0.135)	0.190 (0.281)	0.133 (0.453)	0.146 (0.412)	0.083 (0.640)
Social contact	-0.452 (0.007)**	-0.184 (0.298)	-0.251 (0.152)	-0.243 (0.167)	-0.284 (0.103)	-0.054 (0.760)
Swelling in the neck	-0.242 (0.168)	-0.147 (0.408)	0.040 (0.821)	-0.252 (0.150)	-0.039 (0.825)	0.010 (0.954)
Weight loss	-0.128 (0.472)	-0.317 (0.068)	-0.312 (0.072)	-0.186 (0.292)	-0.266 (0.128)	-0.486 (0.004)**
Problems with wound healing	-0.287 (0.100)	-0.254 (0.148)	-0.303 (0.081)	-0.240 (0.172)	0.139 (0.433)	0.032 (0.856)
Neurological problems	-0.224 (0.203)	-0.340 (0.049)*	-0.317 (0.068)	-0.151 (0.393)	-0.209 (0.236)	-0.061 (0.733)

## Discussion

This study examined the QoL and psychological distress status of survivors of NPC. For most populations, the male-to-female ratio for NPC is approximately 2-3:1. However, the incidence of NPC in high-risk populations peaks at 45-59 years of age [12]. Our results correspond with these findings, as most of our patients were men, with 20 men (58.8%) and 14 women (41.2%), and the patients had a mean age of 49.88 ± 2.27 years.

Radiotherapy's long-term side effects contribute to a worse level of QoL [13]. In our study of NPC survivors, the prevalence of post-treatment symptoms was notably high, indicating significant long-term impacts on QoL. The most common symptoms were oral dryness in 28 patients (82.4%), followed by hearing loss in 25 patients (73.5%), difficulty swallowing in 19 patients (55.9%), fatigue in 17 patients (50.0%), visual disturbances in 17 patients (50.0%), dry nose in 15 patients (44.1%), nasal congestion in 13 patients (38.2%), and mouth ulcers, the least common symptom, in three patients (8.8%). These percentages are considered high when compared to those in a study published in China [9], wherein the percentage of these symptoms was as follows: “mouth dryness or sores” in 38 patients (17.59%), "decline in hearing” in 39 patients (18.06%), “difficulty swallowing” in 25 patients (11.58%), “fatigue” in 40 patients (18.52%), and “nasal dryness or congestion” in 30 patients (13.89%). These differences may be attributed to the potential aggressiveness or variation in treatment protocols, differences in follow-up care, and the baseline characteristics of our patients. However, persistent fears of disease recurrence were also higher in our population compared with the previous study, with nine patients (26.5%) in our study versus 40 patients (18.52%) in the previous study, reflecting the ongoing psychological burden on survivors.

In a previous retrospective study conducted in Jeddah, Saudi Arabia, 25 survivors of NPC were asked to complete the EORTC QLQ-HN35 to assess their QoL. The results showed that poor social interaction was a significant concern in these patients [6]. Conversely, in our study, the highest mean values for the EORTC QLQ-C30 functional scores were for “role functioning” (81.37 ± 29.23), followed by “emotional functioning” (80.39 ± 20.40), “physical functioning” (78.43 ± 20.24), “cognitive functioning” (75.00 ± 26.35), and “social functioning” (75.00 ± 27.60).

A study conducted in China from 2011 to 2013 included 216 survivors of NPC and assessed their QoL and psychological distress status using the EORTC QLQ-C30 and HADS. The study showed that, while the prevalence rates of depression, anxiety, dry mouth, fatigue, and hearing decline were high in these individuals and persisted for many years after exposure to radiotherapy, the mean “global health status/QoL” was 74.21 ± 14.92, which is considered good when compared to the highest possible score of 100 [9]. Our findings are consistent with those of this study, as abnormal borderline depression, borderline anxiety, and anxiety were identified in our patients, with post-treatment symptoms remaining chronic for the majority of survivors. Nevertheless, the mean score for “global health status/QoL” was 63.97 ± 22.54 for survivors of NPC.

A retrospective study conducted in 2021 at the Hospital of Fujian Medical City included 232 patients and examined factors influencing the psychological status of patients with NPC. It observed that patients experienced an increase in the incidence and severity of anxiety and depression before, during, and after radiotherapy. Specifically, in the fourth week following radiotherapy, the insidious number of anxiety cases was 156 (67.2%), and that of depression was 130 (56%) [14]. In our study, abnormal borderline depression, borderline anxiety, and anxiety were identified in five patients (14.7%), four patients (11.8%), and two patients (5.9%), respectively. This variation may be related to the fact that our participants were required to be cancer-free for at least one year. By selecting patients who had completed treatment and achieved cancer-free status, we had a higher likelihood of reducing the emotional impact of treatment.

In a study conducted in Taiwan, researchers surveyed 356 survivors of NPC to evaluate the QoL of those who had been cancer-free for a duration exceeding two years. They found that survivors with higher educational levels, higher yearly household incomes, and those who had received more advanced radiotherapy treatment had better QoL [15]. Furthermore, another study concluded that socioeconomic status emerged as the most influential factor associated with the health-related QoL of NPC survivors [16]. In the present study, most of the patients had a household income of ≤5,000 SR (18 patients; 52.9%), which could be a contributing factor to the disease burden.

Furthermore, the mean score for “global health status/QoL” was significantly lower in patients with comorbidities compared to those without (p = 0.039), which may underscore the additional burden that comorbidities contribute to QoL.

Strengths and limitations

To our knowledge, no such study has been conducted using these scales in KSA. Although a similar study was previously conducted in Jeddah, it used only one scale, which was an older version, and had a smaller sample size. However, a limitation of this study was the small sample size.

## Conclusions

A significant number of survivors of NPC experienced borderline depression and anxiety. The global health status/QoL was low in advanced tumor stages. Additionally, the mean global health status/QoL was low in the presence of certain factors, including coexisting comorbidities and treatment-related side effects. However, the overall mean global health status/QoL was promising for NPC survivors. Based on our results, we encourage researchers in KSA to conduct similar studies with larger sample sizes and across multiple centers. We also anticipate that our study will guide future investigations on the long-term outcomes of NPC treatment; screening for anxiety and depression before, during, and after treatment to facilitate more timely interventions; and the provision of symptomatic treatment for therapeutic side effects, particularly oral dryness, which was widely observed in our patient cohort, with the mean global health status/QoL score being significantly lower in patients with oral dryness than in those without.
